# Three-dimensional lower limb kinematics and kinetics in femoroacetabular impingement syndrome (FAIS) patients with and without borderline developmental dysplasia of the hip (BDDH) during level walking

**DOI:** 10.1186/s12891-025-08727-4

**Published:** 2025-05-16

**Authors:** Yuang Hao, Shuang Ren, Yichuan Zhu, Tong-Chuan He, Xin Miao, Yan Xu

**Affiliations:** 1 School of Sports Medicine and Rehabilitation, Beijing Sport University, Beijing, China; 2 Department of Sports Medicine, Institute of Sports Medicine of Peking University, Peking University Third Hospital, Beijing, China; 3 Beijing Key Laboratory of Sports Injuries, Beijing, China; 4 Engineering Research Center of Sports Trauma Treatment Technology and Devices, Ministry of Education, Beijing, China; 5 Molecular Oncology Laboratory, Department of Orthopaedic Surgery and Rehabilitation Medicine, The University of Chicago Medical Center, Chicago, America USA; 6 Institute of Sports Medicine, Beijing Key Laboratory of Sports Injuries, Peking University Third Hospital, 49 North Garden Road, Haidian District, Beijing, 100191 China

**Keywords:** Femoroacetabular impingement syndrome, Biomechanics, Gait

## Abstract

**Purpose:**

The impact of femoroacetabular impingement syndrome (FAIS) on gait has been reported; however, no studies have documented the effects of Borderline Developmental Dysplasia of the Hip (BDDH) combined with FAIS on gait. This study aimed to evaluate the kinematic and kinetic abnormalities of the lower extremities in patients with combined FAIS and BDDH during level walking.

**Methods:**

A total of 42 participants were included, consisting of 14 patients with FAIS + BDDH, 14 with isolated FAIS and 14 healthy controls. Full-cycle kinematic and kinetic data were collected via motion capture and force plates. Gait analysis was performed in three planes (sagittal, coronal and transverse) for the hip, knee, ankle and pelvis joints. The range of motion (ROM), kinematics and kinetics were compared across the three groups.

**Results:**

Compared with isolated FAIS patients, FAIS + BDDH patients presented a significantly greater hip flexion angle during terminal stance (*P* < 0.05). Moreover, the hip abduction moment was significantly reduced in the loading response and midstance phases in FAIS + BDDH patients (*P* < 0.05). The knee extension moment was significantly reduced during terminal stance in both FAIS groups (*P* < 0.05). The ankle dorsiflexion angle was significantly greater during midstance in FAIS + BDDH patients than in healthy controls, with concomitant reductions in the ankle dorsiflexion moment (*P* < 0.05). No significant differences were found in the range of motion (ROM) of the pelvis or hip joints and hip moment arm among the three groups (*P* > 0.05).

**Conclusion:**

Compared with patients with isolated FAIS, patients with FAIS combined with BDDH exhibit a gait pattern characterized by biomechanical defects of the hip joint similar to developmental dysplasia of the hip (DDH), increased knee stiffness, and compensatory alterations in the ankle joint.

**Level of evidence:**

V.

## Background

Femoroacetabular impingement syndrome (FAIS) is a morphological abnormality of the hip joint that is commonly observed in young and active adults [1–3]. In a healthy hip joint, sufficient femoral head‒neck offset helps prevent impingement between the femoral neck and the pelvis. Surgical intervention is a commonly employed treatment for FAIS. Existing evidence demonstrates that, in the short term, surgical procedures can lead to significant improvements in joint function and a reduction in pain, thereby enhancing overall clinical outcomes [1, 4]. Borderline developmental dysplasia of the hip (BDDH) refers to a condition falling between a normal hip and adult developmental dysplasia of the hip (DDH) [5]. BDDH is typically defined as an acetabular lateral center-edge angle (LCEA) between 18 and 25 degrees [6, 7]. Furthermore, BDDH combined with hip joint instability is more likely to result in failure during arthroscopic treatment [8]. BDDH has gained considerable clinical attention in recent years [9]. A systematic review reported that the failure rate of arthroscopic surgery in FAIS patients with combined BDDH (FAI + BDDH) is 14.1% [10].

Abnormal hip morphology or movements beyond the physiological range can result in repetitive low-impact loading, particularly during combined movements in the sagittal, frontal, and transverse planes, such as hip flexion, adduction, and internal rotation [1, 3]. Previous studies have reported abnormal gait patterns in patients with DDH [11–13], with insufficient acetabular coverage identified as a primary reason contributing to these abnormalities. However, the underlying reasons for poor clinical outcomes in patients with BDDH remain poorly understood. The poor prognosis in FAI + BDDH patients may be attributed to preoperative abnormal biomechanical alterations in the lower limbs, which may further increase the load on the hip joint. Therefore, the aim of this study was to thoroughly investigate the biomechanical characteristics and alterations in the lower limbs of FAIS + BDDH patients compared with those of isolated FAI patients and healthy controls through gait analysis, with the goal of elucidating the potential impact of BDDH on FAIS patients and providing more effective guidance for rehabilitation strategies and clinical interventions. We hypothesized that gait abnormalities in FAIS + BDDH patients would be more pronounced than those in isolated FAIS patients.

## Methods

### Participants

After institutional review board approval, patients who underwent hip arthroscopy from March 2024 to November 2024 at our institute were selected for gait analysis. The inclusion criteria included patients who were diagnosed with FAIS on the basis of their clinical symptoms and radiographic findings [14]. The exclusion criteria were as follows: (1) previous lower limb surgery [15]; (2) concomitant hip conditions, including hip osteoarthritis (OA) with a Tönnis grade > 1, avascular necrosis, Legg-Calvé-Perthes disease, osteoid osteoma, synovial chondromatosis, pigmented villonodular synovitis, and DDH (LCEA < 18°); (3) lower extremity injuries within the past month; (4) other forms of arthritis, diabetes, or heart disease that limit daily activities; and (5) pincer-type FAIS (LCEA > 40°).

### Data collection

Patient demographic characteristics, including age at surgery, sex, affected side, height, weight, body mass index (BMI) and duration of symptoms, were recorded.

Radiographic examinations were performed preoperatively to obtain an alpha angle (AA) in the Dunn view, lateral center edge angle (LCEA) and Tönnis grade in the anteroposterior (AP) view, as described in previous studies [16–18].

All enrolled patients underwent preoperative MRI with a 3.0 T scanner. Fat-saturated proton density (FSPD) sequences and T2-weighted sequences were performed in the axial, coronal, and oblique sagittal planes, respectively. A previously validated, semiquantitative, MRI-based scoring system (scoring hip osteoarthritis with MRI [SHOMRI]) was used to assess abnormalities in the articular cartilage and labrum of the hip joint separately [19]. The SHOMRI system has been previously used in assessments of hip joint abnormalities in patients with hip OA [20, 21].

### Self-reported outcomes

Patient-reported outcomes (PROs), including the visual analog pain scale (VAS), modified Harris hip score (mHHS) and International Hip Outcome Tool, 12-component form (iHOT-12), were used to assess hip perceived function [22–24]. VAS, mHHS, and iHOT-12 scores were routinely collected preoperatively via questionnaires.

### Gait analysis

Gait analysis was conducted preoperatively. The participants were required to wear fitted swimming trunks and remain barefoot during testing. A total of 37 reflective markers were attached to the participants, which were placed on the lateral and medial malleoli, heel, midpoint of the second metatarsophalangeal joint, first metatarsophalangeal joint, fifth metatarsophalangeal joint, lower one-third and upper one-third of the tibia, lateral lower one-third of the leg, tibial tuberosity, medial and lateral femoral condyles, anterior thigh, lateral thigh, anterior superior iliac spine, posterior superior iliac spine, highest point of the iliac crest, acromion, and right scapula. All patients and healthy participants were marked by the same tester (H.Y.A.) to ensure that the testing results were not affected by intertester variability. Prior to testing, the participants were allowed to walk barefoot at their self-selected comfortable speed along a walkway to acclimate to the testing environment. Afterward, a static calibration trial was recorded. At the start of the test, the participants began walking from a premeasured starting point, ensuring that one foot unintentionally stepped on the first force plate while the other foot stepped on the second force plate. A successful trial was characterized by each foot making contact with a force plate, and participants were instructed to walk at their self-perceived comfortable speed along the testing walkway, with three valid data collections performed. Kinematic parameters of the lower limbs during walking were captured via an 8-camera infrared high-speed motion capture system (Vicon, Nexus, T40, UK) at a sampling frequency of 100 Hz. Ground reaction force during walking were collected via two three-dimensional force plates (AMTI, BP400600, USA) at a sampling frequency of 1000 Hz.

We routinely asked patients about their hip pain scores during the testing process, and none of the patients experienced significant pain (VAS > 2) during the gait.

### Data reduction and analysis

All kinematic and kinetic data were processed via Visual3D software (C-Motion, USA). All three-dimensional coordinates of the markers were smoothed using a Butterworth low-pass filter with a cutoff frequency set at 10 Hz. The kinetic data was smoothed using a Butterworth low-pass filter with a cutoff frequency set at 100 Hz. The moment when the vertical ground reaction force exceeded 20 N was defined as the foot contact, whereas the moment when it fell below 20 N was designated the foot-off.

Lower limb segment coordinate systems were established on the basis of the positions of the markers. The hip joint center was calculated according to the methodology outlined by Bell [25]. The center of rotation for the knee joint was defined as the midpoint between the medial and lateral femoral condyles, and the center of rotation for the ankle joint was established as the midpoint between the medial and lateral malleoli. Three-dimensional angles for the pelvis, hip, knee and ankle joints were calculated via the Euler angle method, and three-dimensional moments for the hip, knee and ankle joints were computed via inverse dynamics.

**Fig. 1:**
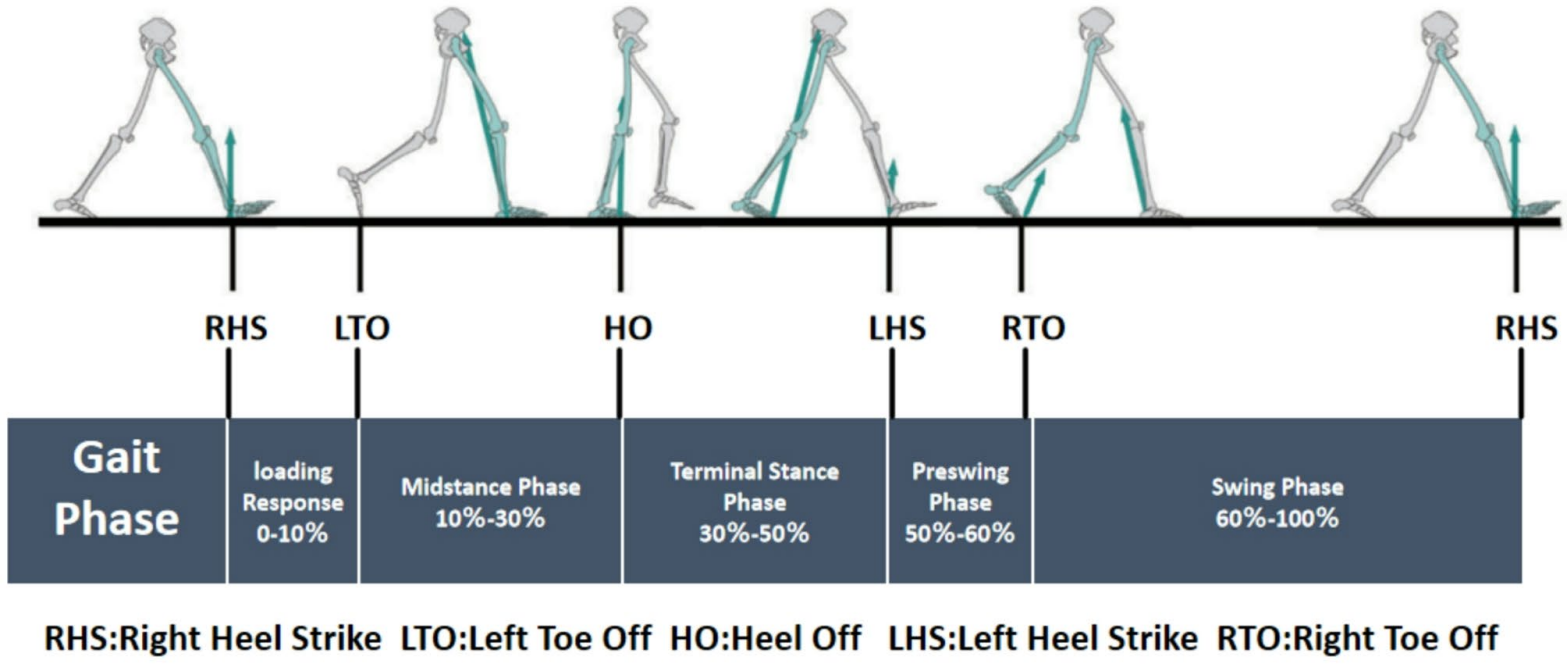
Gait cycle: Subphases in a gait cycle

The joint moment presented in this study is classified as an internal moment. The kinetic parameters included the normalized three-dimensional moment for the hip, knee and ankle joints during the entire gait cycle. The kinematic parameters included normalized three-dimensional angles for each joint throughout the entire gait cycle. The ground reaction forces were standardized as multiples of body weight (BW); The moments were standardized as multiples of height×weight, expressed in units of BW×BH. For each of the kinematic and kinetic components, 101 discrete points corresponding to the 0–100% gait phase at a 1% interval were normalized via a cubic spline for statistical analysis. As shown in Fig. 1, a complete gait cycle includes the entire action phase from heel strike to the subsequent heel strike of the same foot.

The range of joint motion is defined as the difference between the peak and trough angles during the gait cycle, while the hip joint moment arm is defined as the vertical distance from the center of the hip joint to the ground reaction force.

### Statistical analysis

One-way ANOVA was conducted to analyze the differences in demographic variables, walking speed and joint range of motion. A post hoc analysis of covariance with Bonferroni correction (*p* < 0.0167) between every two groups was performed. The normality of the data was tested using the Shapiro-Wilk normality test. Independent sample t tests and Mann-Whitney U signed-rank tests were used to compare the differences in radiological variables. Qualitative data were analyzed via either a chi-square test or Fisher’s exact test. The level of statistical significance was set at *P* < 0.05. The kinematic and kinetic waveforms of the hip, knee, and ankle were compared among the control group (the random side of the healthy control group), FAIS group (involved side), and FAIS + BDDH group (involved side) using Statistical Parametric Mapping (SPM). All statistical analyses were performed in MATLAB software (version: 2016b, MathWorks, USA). For the SPM analysis, Post hoc comparisons between every two groups were performed using independent samples t-tests with correction (*p* < 0.0167) to identify specific regions of significant differences within the waveforms. The statistical differences in SPM are expressed as the differences at each individual point within the interval. Statistical differences in joint moments after 60% of the gait cycle were disregarded because this phase corresponds to the swing phase, during which there are no ground reaction forces. As joint moments cannot be calculated using inverse dynamics during this phase, we chose to disregard their statistical differences. This approach was taken to align the kinetic data with the kinematic data of the gait cycle.

## Results

**Fig. 2:**
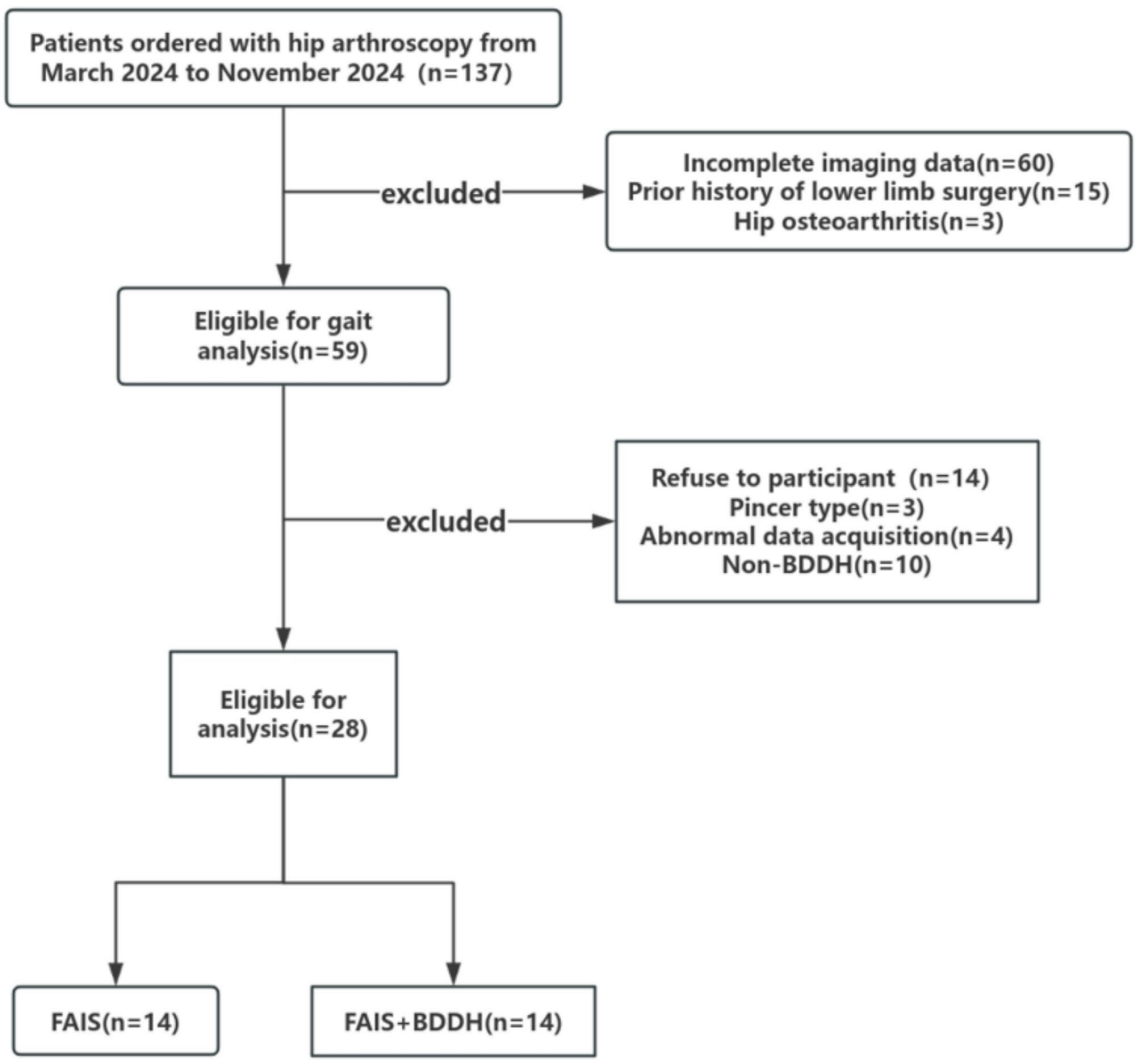
Flow chart of patient selection

The total sample of 42 subjects achieves 99% power to detect differences among the means versus the alternative of equal means using an F test with a 0.05 significance level. As shown in Fig. 2, a total of 28 volunteers aged 18–50 years who were diagnosed with cam-type FAIS and scheduled for hip arthroscopy were recruited. Of these, 14 participants were diagnosed with BDDH (LCEA: 18° < LCEA < 25°). All participants were recruited from the admission records of a sports medicine physician (Y.X.). Additionally, 14 asymptomatic control participants with no history of hip or groin pain or lower extremity surgery were recruited from the university community. The study was approved by the institutional medical research ethics committee, and written informed consent was obtained from all participants.

As shown in Table 1, no significant differences were observed in the baseline data regarding sex, age, BMI, AA, duration of symptoms, patient-reported outcomes, or imaging scores (*p* > 0.05). The lateral center-edge angle in the BDDH group was significantly smaller than that in the FAIS group (*p* < 0.001).

**Table 1:** Demographics, walking speeds, and outcome measures for the study groups

		FAIS + BDDH(*n* = 14)	FAIS(*n* = 14)	Healthy Control(*n* = 14)	*P* Value
BMI($\bar x \pm s$, kg/m²)	20.59 ± 1.5	21.35 ± 3.4	22.81 ± 2.89	0.10	
Age($\bar x \pm s$, y)		32.14 ± 8.58	32.5 ± 6.64	28.71 ± 5.97	0.31
Walking speed(x ± s, m/s)		1.12 ± 0.14	1.17 ± 0.16	1.24 ± 0.13	0.078
Sex(Male: Female)		3: 11	2: 12	6: 8	0.20
α angle		62.49 ± 7.84	68.08 ± 12.25	/	0.16
Lateral Center-Edge Angle		22.71 ± 2.23	34.26 ± 4.81	/	**<0.001**
Duration of symptoms, months($\bar x \pm s$, m)		12.0(8.0, 33.0)	12.0(9.0, 39.0)	/	0.83
VAS		3.07 ± 1.77	3.86 ± 1.61	/	0.23
Modified Harris Hip Score		72.64 ± 12.85	69.71 ± 12.57	/	0.55
International Hip Outcome Tool-12		44.03 ± 14.71	46.16 ± 13.69	/	0.70
Scoring Hip Osteoarthritis with MRI -Total score		7.86 ± 2.83	6.79 ± 2.94	/	0.34
Scoring Hip Osteoarthritis with MRI -Labrum		11.71 ± 3.33	10.21 ± 3.66	/	0.27

Note: an indicates a comparison with FAIS+**BDDH**, *p* < 0.05; b indicates a comparison with **FAIS**, *p* < 0.05; all pairwise comparisons were adjusted via the Bonferroni correction

### Hip

#### Hip joint in the sagittal plane

**Fig. 3:**
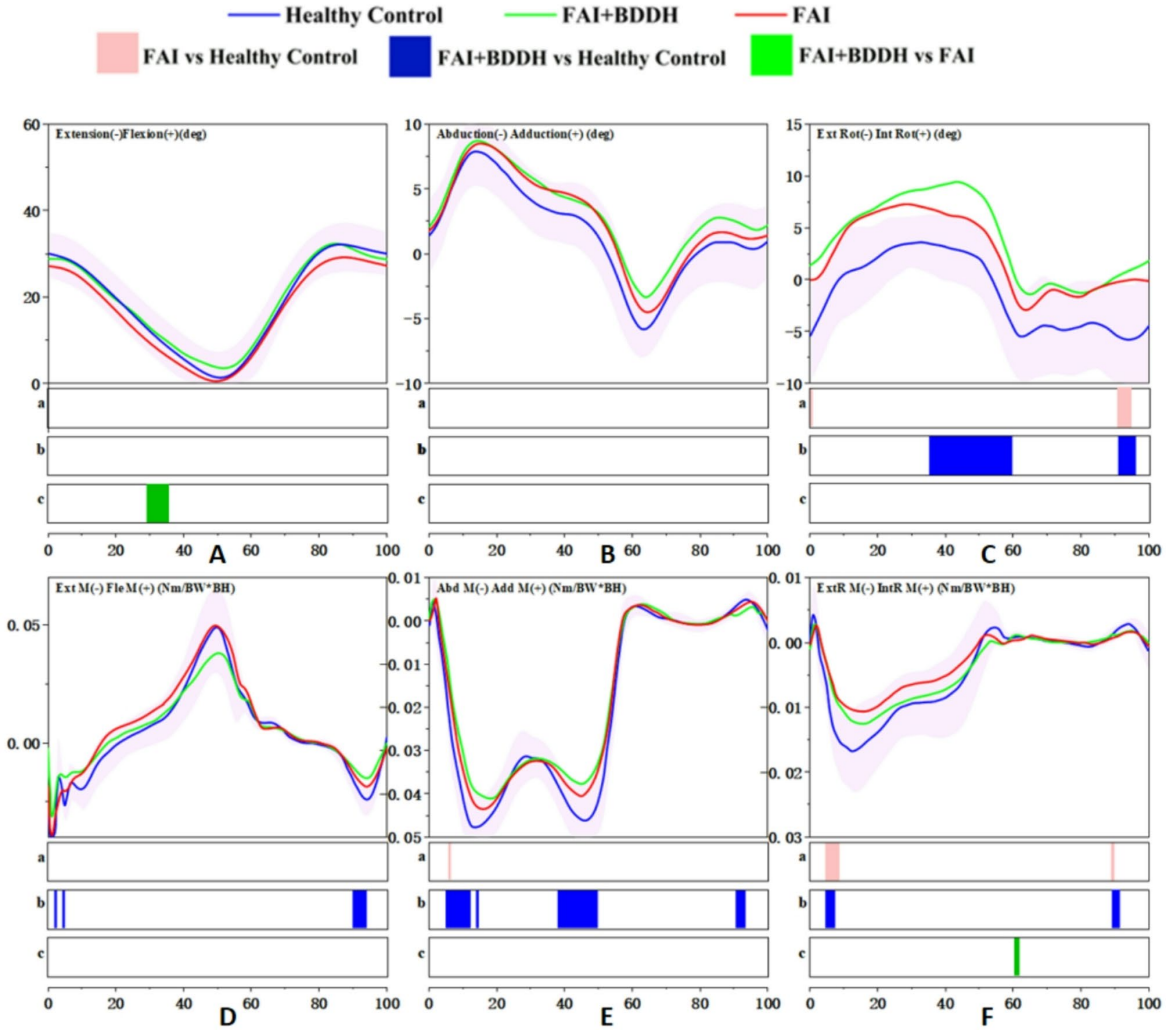
Full-cycle hip joint angle and moment. SPM results are displayed below the figure and indicate significant (*p* < 0.05) differences between (**a**) FAI and Healthy Control, (**b**) FAI + BDDH and Healthy Control, and (**c**) FAI + BDDH and FAI. The statistical differences in joint moments occurring after 60% of the gait cycle should be disregarded

Angle: During the terminal stance phase (29-35%), the hip flexion angle in FAI + BDDH patients was significantly greater than that in patients with isolated FAIS (*p* < 0.05) (Fig. 3A).

Moment: During the loading response phase (2%, 4%), the moment of hip extension in FAI + BDDH patients was significantly smaller than that in healthy controls (*p* < 0.05) (Fig. 3D).

#### Hip joint in the coronal plane

Angle: No statistically significant differences were detected in the hip joint angle in the coronal plane among FAI + BDDH patients, isolated FAIS patients and healthy controls (*p* > 0.05) (Fig. 3B).

Moment: During the loading response, midstance and terminal stance phases (3-14%, 16%, 40-51%), the hip abduction moment in FAI + BDDH patients was significantly smaller than that in healthy controls (*p* < 0.05). Similarly, during the loading response phase (5-6%), the hip abduction moment in isolated FAIS patients was significantly smaller than that in healthy controls (*p* < 0.05) (Fig. 3E).

#### Hip joint in the transverse plane

Angle: During the midstance, terminal stance and terminal swing phases (35-60%, 14%, 40-51%, 91-96%), the internal rotation angle of the hip in FAI + BDDH patients was significantly greater than that in healthy controls (*p* < 0.05). In isolated FAIS patients, the hip external rotation angle was significantly smaller than that in healthy controls (*p* < 0.05) during the loading response phase (0-1%) and the terminal swing phase (91-95%) (Fig. 3C).

Moment: During the loading response phase (5-7%), the hip external rotation moment in FAI + BDDH patients was significantly smaller than that in healthy controls (*p* < 0.05). Similarly, during the loading response phase (5-8%), the hip external rotation moment in isolated FAIS patients was significantly smaller than that in healthy controls (*p* < 0.05) (Fig. 3F).

### Knee

#### Knee joint in the sagittal plane

**Fig. 4:**
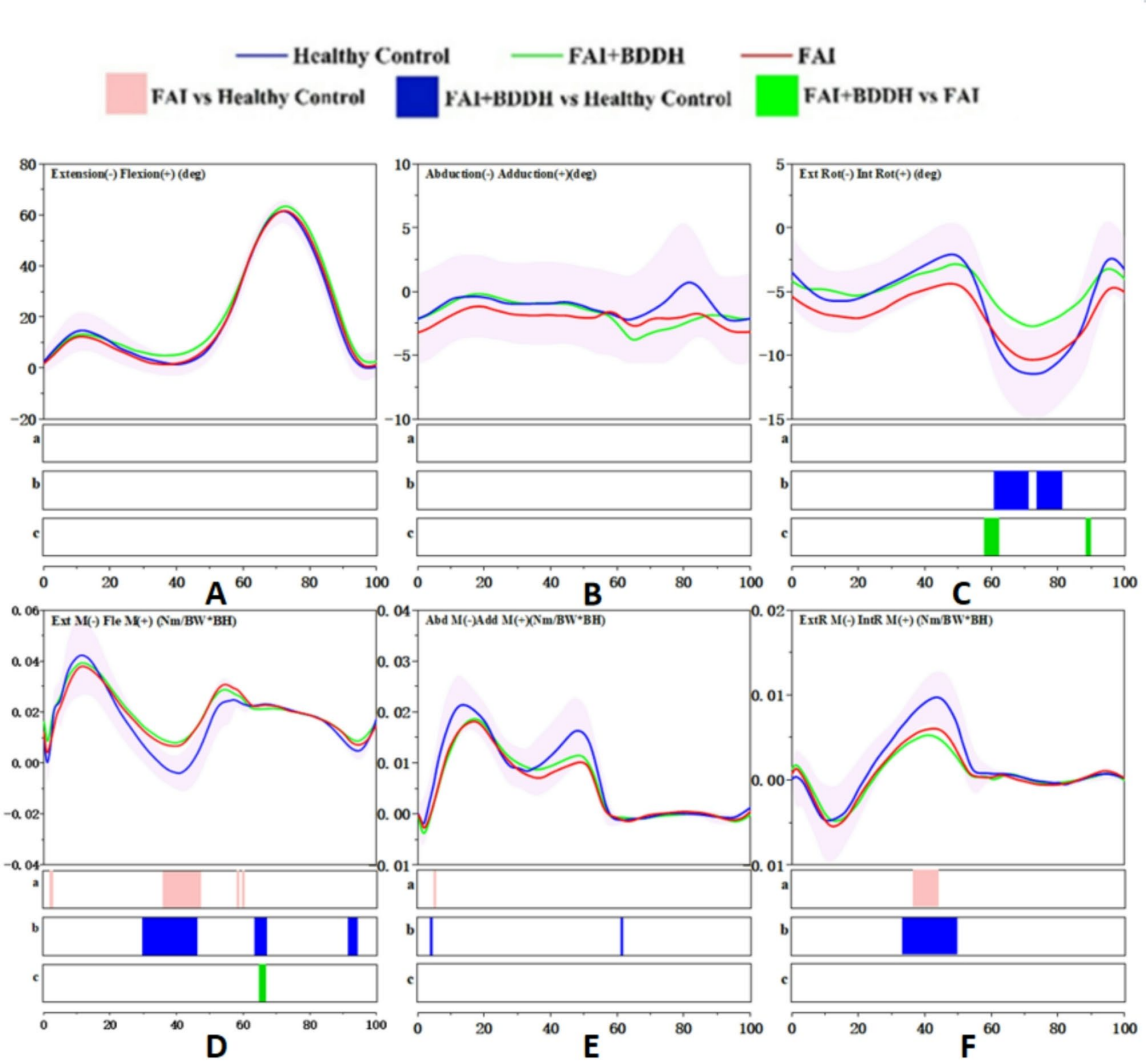
Full-cycle knee joint angle and moment. SPM results are displayed below the figure and indicate significant (*p* < 0.05) differences between (**a**) FAI and Healthy Control, (**b**) FAI + BDDH and Healthy Control, and (**c**) FAI + BDDH and FAI. The statistical differences in joint moments occurring after 60% of the gait cycle should be disregarded

Angle: No statistically significant differences were detected in the knee joint angle in the sagittal plane among FAI + BDDH patients, isolated FAIS patients and healthy controls (*p* > 0.05) (Fig. 4A).

Moment: During the terminal stance phase (30-48%), the knee extension moment in FAI + BDDH patients was significantly smaller than that in healthy controls (*p* < 0.05). Similarly, during the loading response, terminal stance and preswing phases (2%, 32-46%, 58%, and 60%, respectively), the knee extension moment in isolated FAIS patients was significantly smaller than that in healthy controls (*p* < 0.05) (Fig. 4D).

#### Knee joint in the coronal plane

Angle: No statistically significant differences were detected in the knee joint angle in the coronal plane among FAI + BDDH patients, isolated FAIS patients and healthy controls (*p* > 0.05) (Fig. 4B).

Moment: During the loading response phase (6%), the knee adduction moment in FAI + BDDH patients was significantly smaller than that in healthy controls (*p* < 0.05). Similarly, during the loading response phase (6-7%), the knee adduction moment in isolated FAIS patients was significantly smaller than that in healthy controls (*p* < 0.05) (Fig. 4E).

#### Knee joint in the transverse plane

Angle: During the swing phase (74-82%, 61-72%), the knee external rotation angle in FAI + BDDH patients was significantly smaller than that in healthy controls (*p* < 0.05). Additionally, during the preswing phase, swing phase (59-62%, 89-90%), the external rotation angle in isolated FAI patients was significantly larger than that in FAI + BDDH (*p* < 0.05) (Fig. 4C).

Moment: During the terminal stance phase (35-50%), the knee internal rotation moment in FAI + BDDH patients was significantly smaller than that in healthy controls (*p* < 0.05). Additionally, during the terminal stance phase (38-46%), the internal rotation moment in isolated patients was significantly smaller than that in healthy controls (*p* > 0.05) (Fig. 4F).

### Ankle

#### Ankle joint in the sagittal plane

**Fig. 5:**
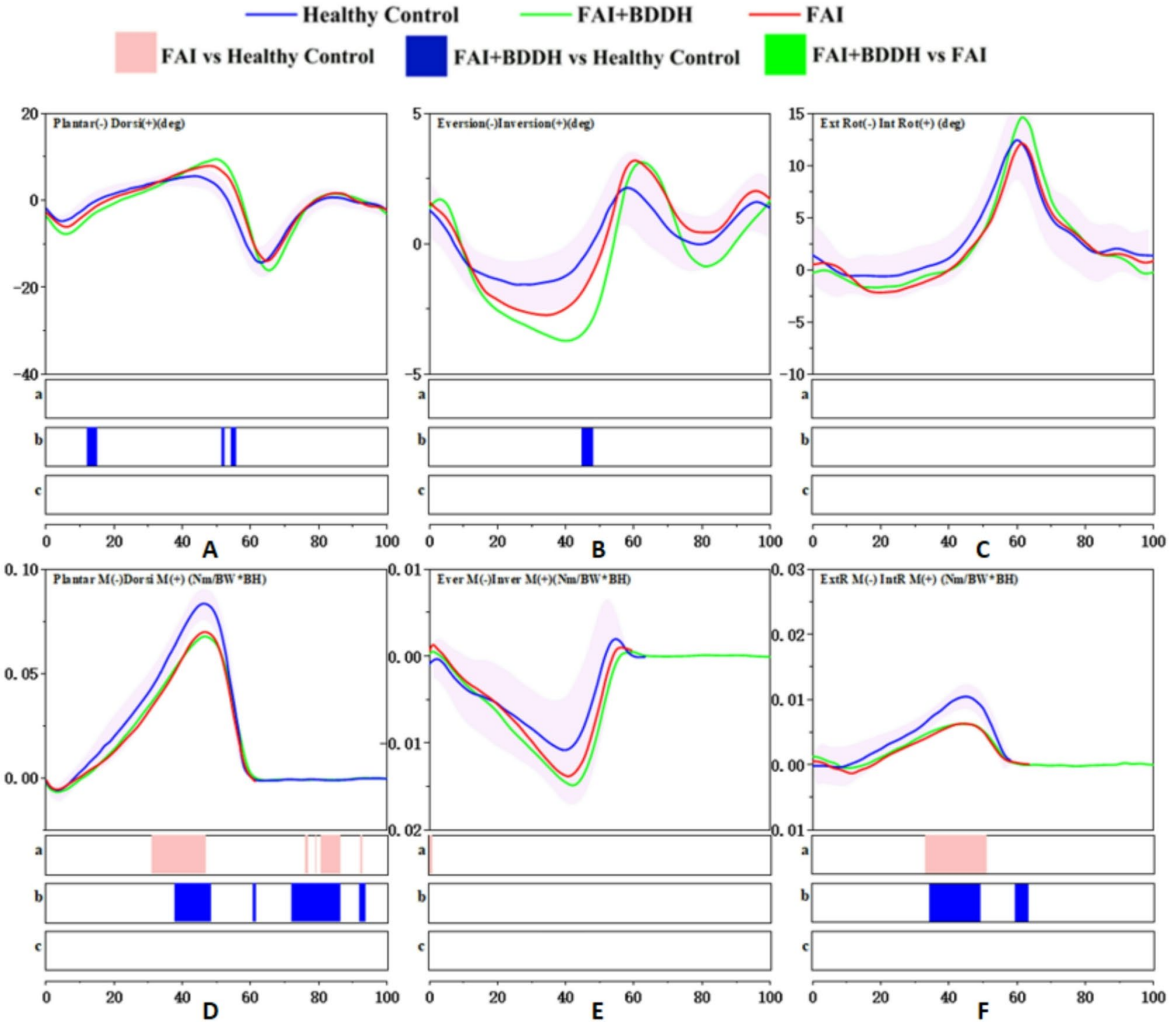
Full-cycle ankle joint angle and moment. SPM results are displayed below the figure and indicate significant (*p* < 0.05) differences between (**a**) FAI and Healthy Control, (**b**) FAI + BDDH and Healthy Control, and (**c**) FAI + BDDH and FAI. The statistical differences in joint moments occurring after 60% of the gait cycle should be disregarded

Angle: During midstance (11-14%) and preswing (52%, 55-56%), the ankle plantarflexion angle in FAI + BDDH patients was significantly greater than that in healthy controls (*p* < 0.05) (Fig. 5A).

Moment: During terminal stance (37-51%), the ankle dorsiflexion moment in FAI + BDDH patients was significantly smaller than that in healthy controls (*p* < 0.05). In isolated FAIS patients, during the terminal stance phase (32-49%), the ankle dorsiflexion moment was significantly smaller than that in healthy controls (*p* < 0.05) (Fig. 5D).

#### Ankle joint in the coronal plane

Angle: During terminal stance (46-48%), the ankle eversion angle in FAI + BDDH patients was significantly greater than that in healthy controls (*p* < 0.05) (Fig. 5B).

Moment: During the loading response phase (0-1%), the ankle inversion moment in FAI + BDDH patients was significantly greater than that in healthy controls (*p* < 0.05) (Fig. 5E).

#### Ankle joint in the transverse plane

Angle: No statistically significant differences were detected in the ankle joint angles in the transverse plane among FAI + BDDH patients, isolated FAIS patients and healthy controls (*p* > 0.05) (Fig. 5C).

Moment: During terminal stance (37-51%), the internal rotation moment of the ankle in FAI + BDDH patients was significantly smaller than that in healthy controls (*p* < 0.05). In isolated FAIS patients, during the terminal stance phase (36-52%), the internal rotation moment of the ankle was significantly smaller than that in healthy controls (*p* < 0.05) (Fig. 5F).

### Pelvis

#### Pelvis in the sagittal plane

**Fig. 6:**
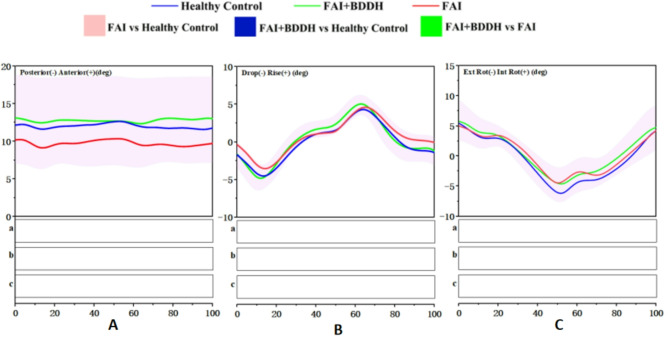
Full-cycle pelvis joint angle. SPM results are displayed below the figure and indicate significant (*p* < 0.05) differences between (**a**) FAI and Healthy Control, (**b**) FAI + BDDH and Healthy Control, and (**c**) FAI + BDDH and FAI

Angle: No statistically significant differences were detected in the pelvis joint angles in the sagittal plane among FAI + BDDH patients, isolated FAIS patients and healthy controls (*p* > 0.05) (Fig. 6A).

#### Pelvis in the coronal plane

Angle: No statistically significant differences were detected in the pelvis joint angles in the coronal plane among FAI + BDDH patients, isolated FAIS patients and healthy controls (*p* > 0.05) (Fig. 6B).

#### Pelvis in the transverse plane

Angle: No statistically significant differences were detected in the pelvis joint angles in the transverse plane among FAI + BDDH patients, isolated FAIS patients and healthy controls (*p* > 0.05) (Fig. 6C).

### GRF

GRF in the Sagittal Plane.

**Fig. 7:**
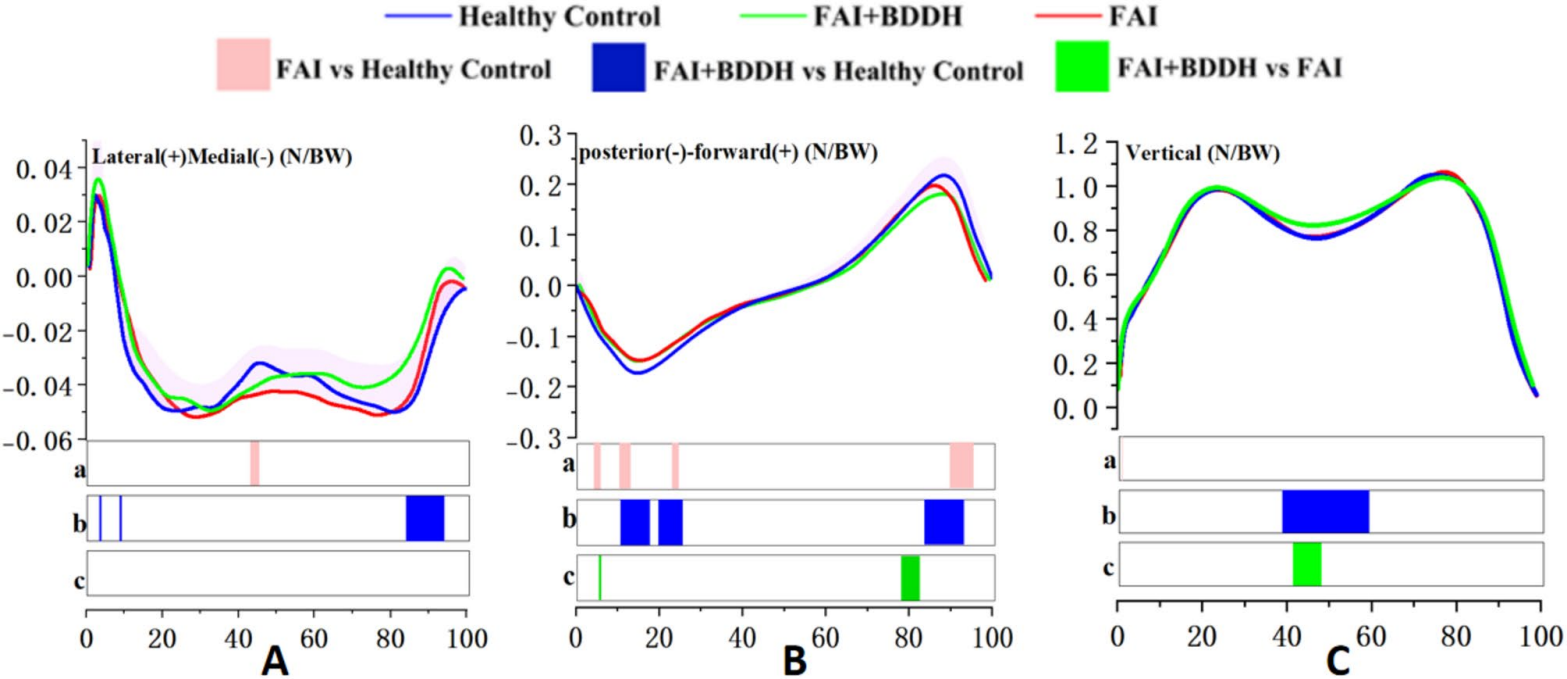
Stand-phase GRF. SPM results are displayed below the figure and indicate significant (*p* < 0.05) differences between (**a**) FAI and Healthy Control, (**b**) FAI + BDDH and Healthy Control, and (**c**) FAI + BDDH and FAI

At 11-18%, 20-25%, and 84-93% of the stance phase, the GRF of FAIS + BDDH was significantly smaller than that of the healthy control group. At 4-6%, 11-14%, 23-24%, and 90-95% of the stance phase, the GRF of FAIS was significantly smaller than that of the healthy control group. Additionally, at 3-4%, 78-83%, and 84-93% of the stance phase, the GRF of FAIS + BDDH was significantly smaller than that of FAIS (Fig. 7B).

#### GRF in the coronal plane

At 5%, 9%, and 83-94% of the stance phase, the GRF of FAIS + BDDH was significantly smaller than that of the healthy control group. At 43-46% of the stance phase, the GRF of FAIS was significantly greater than that of the healthy control group (Fig. 7A).

#### GRF in the vertical plane

At 39-60% of the stance phase, the GRF of FAIS + BDDH was significantly smaller than that of the healthy control group. At 1% of the stance phase, the GRF of FAIS was significantly smaller than that of the healthy control group. Furthermore, at 42-48% of the stance phase, the GRF of FAIS + BDDH was significantly smaller than that of FAIS (Fig. 7C).

No significant differences were detected in the range of motion of the pelvis or hip joints across the three planes among the three groups (*p* > 0.05) (Table 2).

**Table 2:** Range of motion

	FAIS+BDDH(*n* = 14)	FAIS(*n* = 14)	Healthy Control(*n* = 14)	F	*P* Value
HIP ROM sagittal	43.81 ± 3.27	46.11 ± 5.82	46.86 ± 3.79	1.799	0.179
HIP ROM coronal	13.82 ± 2.64	15.35 ± 2.11	15.83 ± 2.81	2.384	0.106
HIP ROM transverse	18.02 ± 3.61	20.99 ± 8.77	16.23 ± 4.99	2.118	0.134
PELVIS ROM sagittal	4.82 ± 1.34	5.22 ± 1.94	4.32 ± 0.87	1.362	0.268
PELVIS ROM coronal	10.97 ± 2.31	10.66 ± 2.37	12.07 ± 2.65	1.28	0.29
PELVIS ROM transverse	15.08 ± 3.92	16.45 ± 12.05	14.56 ± 4.34	0.224	0.8

Note: an indicates a comparison with FAIS+**BDDH**, *p* < 0.05; b indicates a comparison with **FAIS**, *p* < 0.05; all pairwise comparisons were adjusted via the Bonferroni correction

**Fig. 8:**
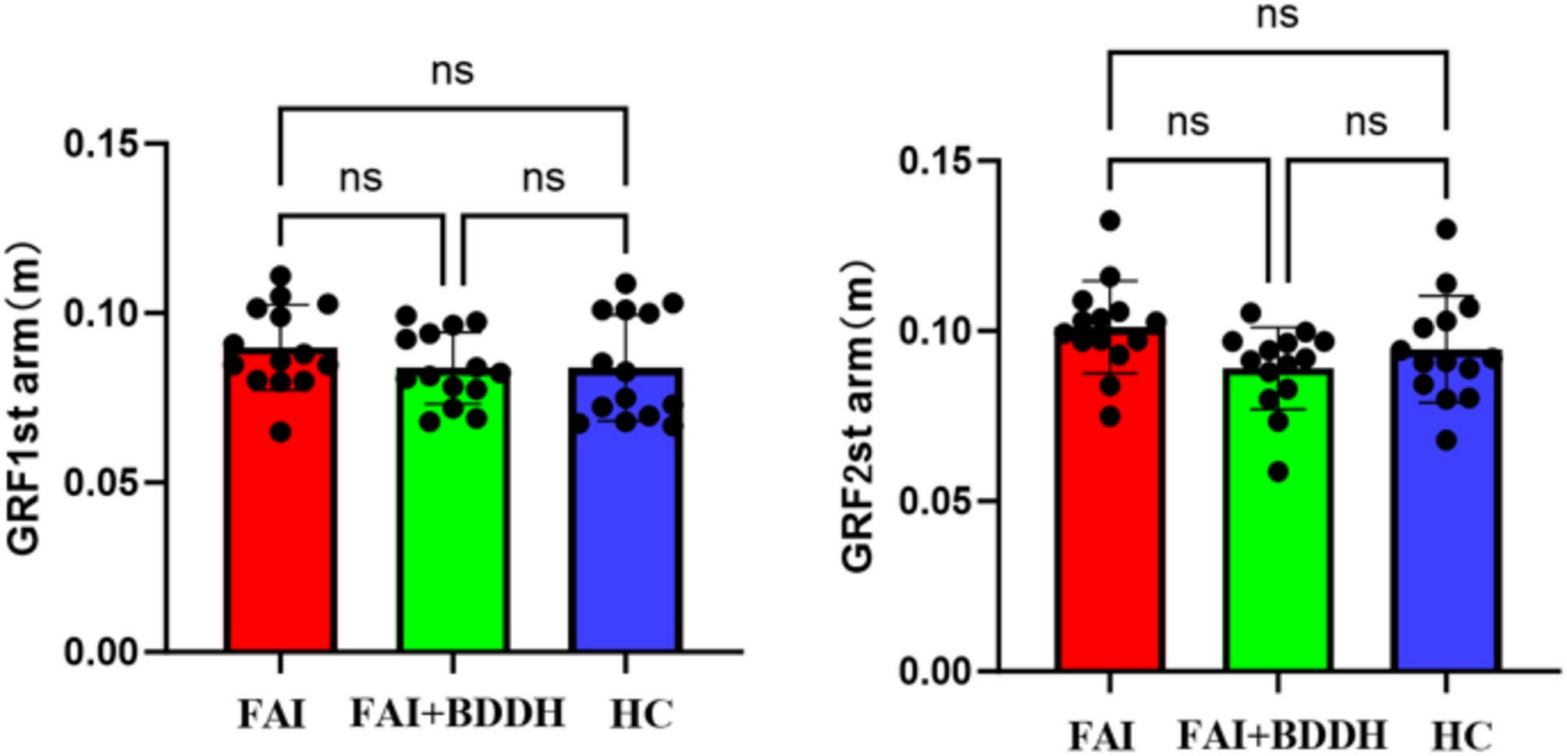
The hip joint moment arm at the first and second peaks of the vertical ground reaction force Note: all pairwise comparisons were adjusted via the Bonferroni correction. HC: Healthy Control

No significant statistical differences in the hip joint moment arm at the first and second peaks of the vertical ground reaction force among the three groups (*p* > 0.05) (Fig. 8).

## Discussion

Our study revealed that patients with FAIS combined with BDDH presented more compensatory biomechanical characteristics during gait than did those with isolated FAIS, including biomechanical defects of the hip joint, similar to DDH, stiffer knee joints, and compensatory alterations in the ankle joints. To the best of our knowledge, this is the first study to evaluate in vivo kinematics and kinetics in patients with combined FAIS and BDDH during level walking.

In the sagittal plane of the hip joint, we observed reduced hip extension angles during the terminal stance phase in BDDH patients compared with those in isolated FAIS patients. Previous studies have reported reduced hip extension angles in FAIS patients [26–29], which may result from the joint approaching the end range of motion during extension. Increased tension in the hip joint ligaments and iliopsoas tendons can cause high pressure at the anterior–superior junction of the femoral head and neck [30, 31]. To avoid this mechanically stressful and pain-inducing scenario, patients adopt a modified gait pattern. Our study suggests that this abnormal gait pattern may be more pronounced in BDDH patients. Additionally, we found that the hip extension moment was reduced during the loading response in patients with combined BDDH. This decrease may stem from weakened hip abductors, extensors, and flexors, as reported in DDH patients [32], although this has not been confirmed in BDDH patients. Future research should focus on muscle strength assessments in BDDH patients to clarify the biomechanical mechanisms involved. We also observed a significantly reduced hip abduction moment during the loading response and midstance phases in patients with combined BDDH compared with healthy controls. In isolated FAIS patients, this phenomenon was observed only during the loading response. This reduction may be attributed to hip abductor weakness, which has been shown in DDH patients to involve shorter abductor moment arms, weaker abductor muscles [32], and a reduced hip abduction moment [33]. Our findings suggest that similar phenomena may occur in BDDH patients, but further electromyographic studies are needed to validate this conclusion. Patients with combined BDDHs exhibited greater hip internal rotation during the terminal stance and preswing phases. Our findings suggest that reduced osseous coverage may allow BDDH patients to achieve a greater range of hip internal rotation during gait.

Previous studies have reported ipsilateral knee pain (IKP) in FAIS patients [34]. We found that the knees of FAIS + BDDH patients displayed a gait pattern characterized by a combination of “stiffening gait” (extension deficiency and reduced extension moment) and “pivot-shift gait” (extension deficiency, along with reduced extension and internal rotation moment). We also observed significant changes in the rotational angle of patients with FAIS + BDDH, but this change may be limited by the choice of marker placement scheme [35], so we remain cautious about this result.

Our study revealed that the ankle dorsiflexion angle during preswing was significantly greater in FAI + BDDH patients than in healthy controls. Moreover, the ankle joint moment significantly decreased. As the external moment must be counteracted by the internal moment, this finding may indicate ankle muscle weakness in FAI + BDDH patients. Ankle dorsiflexion moment reductions were also observed in isolated FAIS patients. However, we detected increased ankle eversion and a decreased external rotation moment in FAI + BDDH patients, further supporting our conclusion that ankle compensation may be present in these patients during gait. Owing to the cross-sectional nature of this study, we cannot confirm whether this compensation is caused by BDDH or whether it exacerbates symptoms or represents a compensatory mechanism. To our knowledge, no previous studies have examined the dynamic relationship between the ankle and hip joints during gait in BDDH patients. Instability in adjacent joints can profoundly impact hip joint function. Future research should investigate the dynamic interplay between the ankle and hip and evaluate whether ankle-strengthening exercises benefit FAIS patients.

The range of motion results revealed no significant differences between the FAIS combined with BDDH, isolated FAIS, and healthy control groups. These findings suggest that acetabular coverage and Cam deformities may have a limited impact on the joint range of motion during gait. Previous studies have reported a reduction in the pelvic and hip joint sagittal and coronal plane angles [26, 36–38]; however, our study did not observe this phenomenon. This may be due to sample selection, as we did not include Pincer-type FAIS patients in our study. Future research should further investigate the impact of acetabular coverage on joint range of motion during gait.

The ground reaction force (GRF) in the coronal plane primarily reflects the lateral stability of the body [39, 40]. In FAIS + BDDH patients, GRF is significantly reduced, corresponding to a decrease in hip abduction moment. This indicates alterations in coronal plane loading in FAIS + BDDH patients, which may represent an important biomechanical characteristic of BDDH and could be an essential factor contributing to compensations in adjacent joints [39]. Future studies should investigate the biomechanical abnormalities in the coronal plane in FAIS + BDDH patients with a larger sample size. The vertical GRF is the most dominant component of the reaction force during gait, directly reflecting the gravitational load exerted on the ground and the body’s support capacity. During most of the stance phase, the GRF in FAIS + BDDH patients was significantly reduced. Additionally, the vertical force in FAIS patients showed smaller shifts during changes in the center of gravity throughout the gait process. This may be associated with a pain-avoidance mechanism, where patients reduce or lighten vertical loading to alleviate hip joint pain or discomfort. Furthermore, impaired joint perception of changes in the center of gravity may be a characteristic movement pattern specific to FAIS + BDDH patients.

No significant statistical differences in hip joint moment arms were found among the three groups. However, it is clear that the moment arms of BDDH patients are all smaller than those of the FAI and healthy control groups. This may be a significant reason for the reduced abduction moment in BDDH patients. Since our hip joint center was estimated based on the Bell method, future studies could use CT imaging to correct the location of the hip joint center to observe more precise changes in moment arms.

The clinical diagnosis, treatment, and surgical methods for patients with FAI + BDDH remain subjects of controversy [10]. Determining how to optimize the improvement of biomechanical deficiencies in FAI + BDDH patients through surgical interventions or rehabilitation exercises may be critical in delaying the progression of osteoarthritis in patients with FAIS + BDDH.

This study offers novel biomechanical insights into the gait abnormalities of patients with combined FAIS and BDDH, with significant implications for clinical practice. Our findings demonstrate that patients with FAIS and BDDH exhibit more severe biomechanical compensation than those with isolated FAIS, including biomechanical defects of the hip joint similar to DDH, increased knee stiffness, and compensatory alterations in the ankle joint. These findings highlight the need for a multifaceted clinical approach that addresses both the structural and functional impairments characteristic of this patient population.

Furthermore, our study provides a deeper understanding of the dynamic interaction between the hip joint and adjacent joints, such as the knee and ankle, in patients with combined FAIS and BDDH. Given the observed biomechanical interdependencies, future research should focus on exploring the efficacy of joint-specific rehabilitation strategies and evaluating whether strengthening adjacent joints (e.g., the ankle) can mitigate the biomechanical deficits observed in the hip and knee. These insights could lead to the development of more refined, evidence-based therapeutic protocols tailored to this complex patient group.

In conclusion, this study contributes to the growing body of literature on the biomechanical profile of FAIS + BDDH patients, offering valuable evidence that can inform the development of more personalized and effective treatment strategies. By improving the understanding of gait abnormalities and their underlying mechanisms, this research has the potential to enhance clinical outcomes, reduce the risk of joint instability, and improve the overall quality of life for individuals affected by these conditions.

Limitations.

Our study has several limitations. First, we did not match participants for sex, age, BMI, or Self-reported outcomes, which may lower the level of evidence. Second, we investigated only biomechanical changes during level walking, an activity that minimally provokes the hip joint. Further research should incorporate more ecological walking protocols and include more strenuous activities, such as running or stair climbing, to provide a more comprehensive understanding. Third, we did not collect data on static joint range of motion (ROM) and muscle strength, which limits our ability to assess their potential influence on the observed biomechanical changes. Finally, we did not perform pelvic anteroposterior radiographs for the healthy control group.

## Conclusion

Patients with FAIS combined with BDDH exhibit a gait pattern characterized by biomechanical defects of the hip joint similar to those with DDH, increased knee stiffness, and compensatory alterations in the ankle joint compared with those with isolated FAIS.

## Data Availability

All relevant data supporting the conclusions are included within the article and tables. The datasets used and/or analyzed during the current study available from the corresponding author on reasonable request.

## Declarations

### Study design

The Impact of BDDH on the Gait of FAI Patients: A Cross-Sectional Study.

## Abbreviations

FAIS: Femoroacetabular impingement syndrome
BDDH: Borderline developmental dysplasia of the hip
ROM: Range of motion
DDH: Developmental dysplasia of the hip
LCEA: Lateral center-edge angle
OA: Hip osteoarthritis
BMI: Body mass index
AA: Alpha angle
AP: Anteroposterior
FSPD: Fat-saturated proton density
SHOMRI: Scoring hip osteoarthritis with MRI
PROs: Patient-reported outcomes
VAS: Visual analog pain scale
mHHS: Modified Harris hip score
iHOT-12: International Hip Outcome Tool (12-component form
BW: Body weight
SPM: Statistical Parametric Mapping
IKP: Ipsilateral knee pain
ACL: Anterior cruciate ligament
